# Two new fossil genera and species of Cerocephalinae (Hymenoptera, Chalcidoidea, Pteromalidae), including the first record from the Eocene

**DOI:** 10.3897/zookeys.545.6470

**Published:** 2015-12-14

**Authors:** Marcel Bläser, Lars Krogmann, Ralph S. Peters

**Affiliations:** 1 Zoologisches Forschungsmuseum Alexander Koenig, Abteilung Arthropoda, Adenauerallee 160, 53113 Bonn, Germany; 2 Staatliches Museum für Naturkunde, Rosenstein 1, 70191 Stuttgart, Germany

**Keywords:** *Tenuicornus*, *Tenuicornus dominicus*, *Pteropilosa*, *Pteropilosa lailarabanorum*, Miocene, Baltic amber, Dominican amber

## Abstract

Cerocephalinae (Chalcidoidea, Pteromalidae) is a small group of parasitoid wasps characterized by a number of derived diagnostic features. Their hosts are endophytic beetles. So far, 43 species of Cerocephalinae have been described, including one fossil species from the Miocene. In this study, we add two new genera and species from Baltic and Dominican amber to the fossil record. *Tenuicornus dominicus* **gen. et sp. n.** is the second genus described from Dominican amber, and *Pteropilosa lailarabanorum* **gen. et sp. n.**, described from Baltic amber, represents the oldest record of the subfamily, pushing the minimum age of Cerocephalinae back to the Eocene. Diagnostic characters of both species are discussed in comparison with other Cerocephalinae. An updated key to extant and fossil Cerocephalinae is presented.

## Introduction

Cerocephalinae is one of the smaller, yet most distinctive subfamilies of Pteromalidae (Hymenoptera, Chalcidoidea). Cerocephaline wasps are comparatively easy to recognize by a prominence in the intertorular area that can be a longitudinal carina or a tooth-like structure of varying size, by typical wing vein length ratios, and by two spurs on the hind tibia. Including the fossil record, only 43 species of Cerocephalinae in 14 genera have been described (Noyes 2014). All species are assumed to be parasitoids of small endophytic beetles (Bouček 1988), which makes them potentially potent biological control agents. Members of the genera *Cerocephala* and *Theocolax* have hereby proven to be effective against pests like *Stegobium paniceum* Linnaeus, 1758, *Rhyzopertha dominica* Fabricius, 1792 and other crop-feeding beetles (Flinn and Hagstrum 2002, Adarkwah et al. 2014).

Recent studies (Munro et al. 2011, Heraty et al. 2013) support the monophyly of Cerocephalinae, but their phylogenetic placement within Chalcidoidea still poses a problem. Pteromalidae is recognized as a polyphyletic assemblage (e.g., Krogmann and Vilhelmsen 2006, Heraty et al. 2013), but the phylogenetic relations among the pteromalid subfamilies and the remaining Chalcidoidea are unresolved. Among these, Cerocephalinae have not been studied intensively. A phylogeny of the taxa of Cerocephalinae has never been established, and only few taxonomic studies (e.g., Hedqvist 1969, Bouček 1988, 1993, Krogmann 2013) have been published since Gahan’s (1946) first comprehensive revision of the group.

Cerocephalinae stand out as particularly underrepresented in the fossil record, with only one representative from Dominican amber, *Dominocephala vibrissae* Krogmann, 2013, known so far. In this study, two additional fossils representing two new genera and species are described. One of the new genera was found in Dominican amber (same as *Dominocephala vibrissae*), which is estimated to be 20–15 million years old (Iturralde-Vinent and MacPhee 1996). The other genus was found in Baltic amber, which is estimated to be 56–34 million years old (Szwedo and Sontag 2009). The latter taxon now represents the oldest record of the subfamily Cerocephalinae. Both genera are compared to extant members of Cerocephalinae and an updated key to the genera of this subfamily is given.

## Material and methods

**Figure 1.**
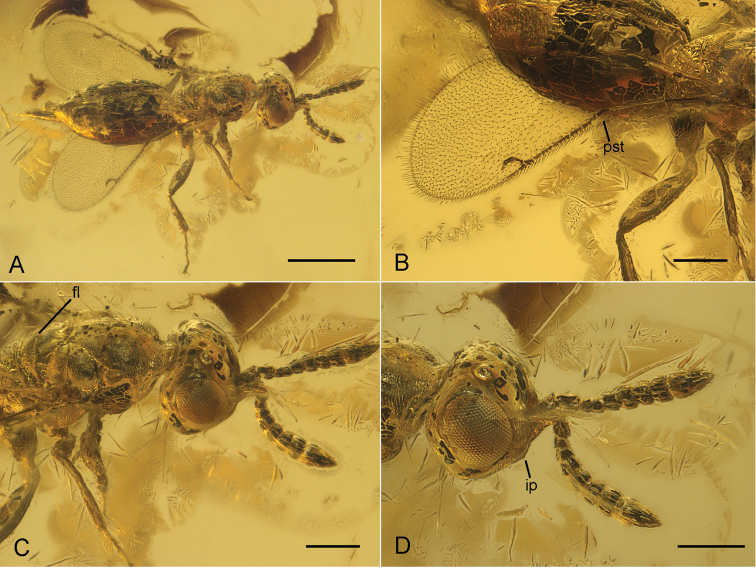
*Pteropilosa lailarabanorum* gen. et sp. n. in dorsolateral view, showing general habitus and diagnostic characters. **A** General habitus **B** Fore wing with distinct pilosity on disc, with comparatively short marginal vein, and without a tuft of erect setae on the parastigma (pst) **C** Head and mesosoma, showing surface sculpture on pro-, and mesonotum, and distinct frenal line (fl) **D** Head and antennae, showing tooth-shaped intertorular prominence (ip) positioned slightly below insertion of antennae; antennae inserted well above ventral margin of eyes, segments 3–6 of the 6-segmented funicle distinctly transverse.Scale: 500 µm (**A**); 200 µm (**B–D**).

**Figure 2.**
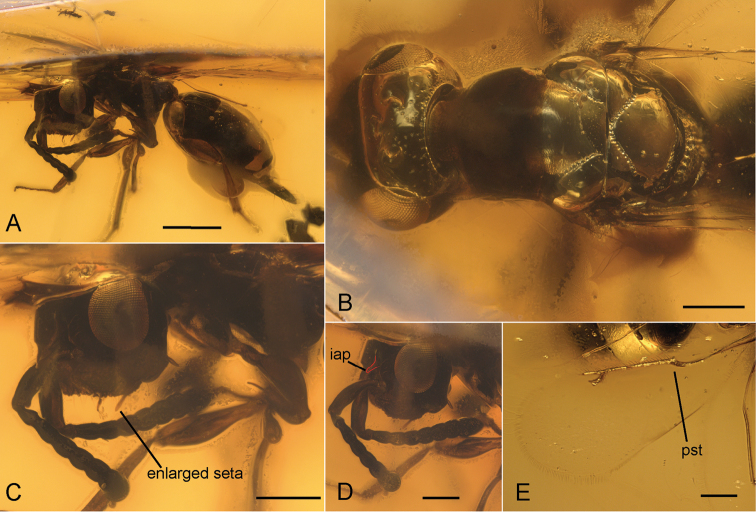
*Tenuicornus dominicus* gen. et sp. n., showing general habitus and diagnostic characters. **A** General habitus in frontolateral view, showing rather long exerted part of the ovipositor **B** Head and mesosoma in dorsal view, mesosoma with polished mesonotum, foveolate notauli, and absent frenal line **C** Head in frontolateral view, with deep facial impression and enlarged setae at each side of lateral margin of the impression **D** Head and antennae in frontolateral view, at slightly different angle than **C**, with nail-shaped inner antennal process (iap) visible and highlighted with a red line; antennae inserted at ventral margin of eyes, with long scape, and 6-segmented, clavate funicle **E** Fore wing without a tuft of erect setae at parastigma (pst) and without pilosity or setal bases on wing disc. Scale: 500 µm (**A**); 200 µm (**B–D**).

Terminology follows Gibson (1997) with additions from Krogmann (2013). Descriptions of surface sculpture follow Harris (1979). Images for Figures 1 and 2 were taken with a Keyence VHX 600 digital microscope using incident light. Images for measurements were taken with a Leica DXM 1200 digital camera attached to a Leica MZ 16 APO microscope and processed using Auto-Montage (Syncroscopy) software. All images were edited with Adobe Photoshop CS3 and figure plates assembled with Adobe Illustrator CS3.

## Systematics

### Subfamily Cerocephalinae

Taxon classificationAnimaliaHymenopteraPteromalidae

Gahan, 1946

#### Diagnosis.

Intertorular area with prominence that can be a carina or tooth-shaped (also referred to as inner antennal process (iap)). Fore wing disc bare or with setae reduced to setal bases (pilose only in *Pteropilosa* gen. n.), with marginal fringe, fore wing always with thickened juncture between marginal vein and submarginal vein (parastigma), marginal vein elongate, postmarginal and stigmal vein shortened, postmarginal vein usually shorter than stigmal vein. Mesonotum with complete notauli. Hind tibia with two spurs.

#### Key to extant and fossil genera of Cerocephalinae

This key is modified after Krogmann (2013) and includes the two new fossil genera described herein.

**Table:** 

1	Antenna with five funicular segments in females and six funicular segments in males	**2**
–	Antenna with six funicular segments in females (Fig. 1D) and seven funicular segments in males	**4**
2	Antenna shortened, with all funicular segments transverse; fore wing without a tuft of erect setae on parastigma (apterous species occur)	***Choetospilisca* Hedqvist, 1969** [USA, Brazil, India]
–	Antenna of normal size, with all funicular segments longer than or about as long as wide; fore wing with or without a tuft of erect setae on parastigma	**3**
3	Fore wing with a tuft of erect setae on parastigma	***Theocolax* Westwood, 1832** (in part) [Cosmopolitan]
–	Fore wing without a tuft of erect setae on parastigma (as in Fig. 2E)	***Acerocephala* Gahan, 1946** (in part) [USA, Samoa]
4	Fore wing disc distinctly pilose (Fig. 1B)	***Pteropilosa* gen. n.** [fossil, Eocene Baltic amber]
–	Fore wing disc bare, setae (if present) reduced to setal bases	**5**
5	Head, pronotum and mesoscutum sculptured; fore wing without a tuft of erect setae on parastigma	**6**
–	Head dorsally and mesoscutum entirely smooth and polished; pronotum usually polished but sometimes partly sculptured; fore wing with or without a tuft of erect setae on parastigma	**10**
6	Mandibles enlarged and conspicuous, at least 1/3 as long as head capsule	**7**
–	Mandibles of normal shape and dimensions, less than 1/3 as long as head capsule	**9**
7	Mandibles without a basal process	***Muesebeckisia* Hedqvist, 1969** [Brazil]
–	Mandibles with a basal process	**8**
8	Mandibles with two distinct teeth	***Gnathophorisca* Hedqvist, 1969** [Brazil]
–	Mandibles with three distinct teeth	***Gahanisca* Hedqvist, 1969** [Brazil]
9	Antenna inserted at level of ventral margin of eye	***Neosciatheras* Masi, 1917** [Seychelles]
–	Antenna inserted much higher than ventral margin of eye	***Sciatherellus* Masi, 1917** [Seychelles]
10	Mandible elongate and conspicuous, 1/3 to 3/4 as long as head capsule	**11**
–	Mandible of normal size, far less than 1/3 as long as head capsule	**13**
11	Head with row of conspicuously elongate setae extending dorsally from the lower facial process; fore wing without a tuft of erect setae on parastigma	***Dominocephala* Krogmann, 2013** [fossil, Miocene Dominican amber]
–	Head without a row of conspicuously elongate setae; fore wing with or without a tuft of erect setae on parastigma	**12**
12	Mandible two-dentate; fore wing with a tuft of erect setae on parastigma	***Paralaesthia* Cameron, 1884** [Panama]
–	Mandible four-dentate; fore wing without a tuft of erect setae on parastigma	***Acerocephala* Gahan, 1946** (in part) [USA, Samoa]
13	Antenna inserted distinctly below level of ventral margin of eye; head in dorsal view parallel-sided	***Theocolax* Westwood, 1832** (in part) [Cosmopolitan]
–	Antenna inserted at or slightly below level of ventral margin of eye; head in dorsal view not parallel-sided	**14**
14	Fore wing with a tuft of erect setae on parastigma	**15**
–	Fore wing without a tuft of erect setae on parastigma	**16**
15	Antenna with all funicular segments transverse, i.e., shorter than wide; propodeum with median carina	***Paracerocephala* Hedqvist, 1969** [Zaire]
–	Antenna with all funicular segments longer than wide or at least as long as wide; propodeum without median carina	***Cerocephala* Westwood, 1832** [Cosmopolitan]
16	Antenna with all funicular segments longer than wide, cylindrical, almost parallel-sided	***Laesthiola* Bouĉek, 1993** [USA]
–	Funicular segments different; most funicular segments shorter than wide or as long as wide	**17**
17	Face deeply impressed, with long setae lateral to the impression of face; intertorular prominence nail-like and positioned distinctly above toruli (Fig. 2C, D)	***Tenuicornus* gen. n.** [fossil, Miocene Dominican amber]
–	Face convex, without long setae lateral to the impression of face; intertorular prominence variable in shape but never nail-like and positioned at level of or below toruli	***Neocalosoter* Girault & Dodd, 1915** [Southern Hemisphere, Neotropics, Nearctic, Philippines]

### Pteropilosa gen. n.

Taxon classificationAnimaliaHymenopteraPteromalidae

http://zoobank.org/224D6EC0-9AB4-41E0-9558-6430B75D62B1

#### Type species.

*Pteropilosa lailarabanorum* sp. n.

#### Material.

Female holotype, preserved in Eocene Baltic amber (56-34 Ma). Holotype deposited in the amber collection of the State Museum of Natural History, SMNS collection number BB-2815.

#### Diagnosis.

Funicle of female 6-segmented with funicular segments 3-6 distinctly transverse, i.e., shorter than wide (Fig. 1D); toruli positioned near upper margin of eyes (upper third of eyes); intertorular prominence tooth-shaped, positioned slightly below level of toruli; shape of head almost round (Fig. 1D). Fore wing pilose (Fig. 1B); marginal vein less than four times as long as stigmal vein; parastigma without a tuft of erect setae (Fig. 1B). Mesosoma sculptured and irregularly imbricate with short and transverse strigulate lines; frenal line present (Fig. 1C).

#### Etymology.

The generic name *Pteropilosa* is composed of two parts. The first being *Ptero*-, which is derived from the old-Greek “pteryx”, meaning “wing”; the last letters -*pilosa* are derived from the Latin “capillosus”, meaning “hairy”. It can be roughly translated as “hairy wings”, referring to the most striking unique feature of this new genus. The generic name is feminine in gender.

### Pteropilosa lailarabanorum sp. n.

Taxon classificationAnimaliaHymenopteraPteromalidae

http://zoobank.org/6C7DCAE6-9192-49F3-B4DC-77A9F0DCF4A3

#### Diagnosis.

See genus.

#### Description.

Female: total body length 1.62 mm; length of head 0.32 mm, of mesosoma 0.64 mm, of metasoma 0.86 mm. Body without metallic luster. *Head*: height 0.35 mm. Shape of head round (globose) and head smooth, without sculpture; no depression on face; mandibles small (not measurable). Eyes rather big and slightly egg-shaped, 0.22 mm high and 0.19 mm wide. Intertorular prominence tooth-shaped and positioned between toruli, but slightly below level of toruli. *Antennae*: inserted well above ventral margin of eyes (in upper third of eyes); scape length 96 µm, pedicel length 48 µm, pedicel rather short and stout; funicle 6-segmented. First funicular segment wider than long, F2 longer than wide. F3-F6 wider than long, distally broadened club-like (F1: length: 33 µm × width: 37 µm; F2: 40 µm × 38 µm; F3: 47 µm × 56 µm; F4: 48 µm × 69 µm; F5: 48 µm × 71 µm; F6: 54 µm × 77 µm); all funicle segments with sensilla. Clava egg-shaped, 0.14 mm long and 81 µm wide. *Wings*: fore wing disc pilose, without fuscous band. Elongate admarginal setae absent. Fore wing length 1.19 mm and width 0.33 mm; submarginal vein 0.42 mm long, marginal vein 0.33 mm and postmarginal vein 76 µm long, stigmal vein curved, 76 µm long; stigma slightly thickened and uncus visible. Hind wing length 0.98 mm and width 0.23 mm, only two hamuli visible. *Legs*: length of hind tibia 0.24 mm, hind femur 0.35 mm, hind trochanter 46 µm, hind coxa 0.15 mm. The other legs cannot be measured due to the position of the specimen within the amber. *Mesosoma*: pronotum, mesoscutum and mesoscutellum completely reticulated. Frenal line clearly visible and foveolate; frenum 0.55 times as long as mesoscutellum. Length of pronotum 0.25 mm, of mesoscutum 0.13 mm, of mesoscutellum 50 µm, of frenum 29 µm. Axillae large, medially connected. Metanotum with distinct foveae; lateral panel of metanotum wide and smooth. Metascutellum thin; not reaching anterior margin of metanotum. Propodeum coarsely reticulated. *Metasoma*: petiole (Mt_1_) small, transverse, hardly recognizable. Metasomal tergites Mt_2_ to Mt_9_ smooth, Mt_2_: 0.23 mm, Mt_3_: 42 µm, Mt_4_: 0.15 mm, Mt_5_: 0.13 mm, Mt_6_: 67 µm, Mt_7_ 0.10 mm, Mt_8/9_: 70 µm. Ovipositor 0.12 mm (top view) exerted beyond end of gaster.

#### Taxonomic remarks.

Following the latest available identification key of Krogmann (2013), *Pteropilosa lailarabanorum* would be determined as *Sciatherellus*. However, there are distinct differences between the two genera. In addition to the pilose fore wings which are unique in *Pteropilosa* (Fig. 1B), *Sciatherellus* differs from *Pteropilosa lailarabanorum* in having six antennal funiculars that broaden distally, with F3-6 distinctly transverse (Fig. 1D). In contrast, no funicular segment in *Sciatherellus* is wider than long. The fore wings of *Sciatherellus* also possess two prominent dark transverse bands that are absent in *Pteropilosa*, and the stigmal vein is straight in contrast to that of *Pteropilosa lailarabanorum*, which is distinctly curved (Fig. 1B). The main differences between *Pteropilosa* and *Neocalosoter* are the pilose wings, the sculptured mesosoma, and the position of the toruli. In *Pteropilosa*, the antennae are inserted distinctly above the lower margin of the eyes, at around 2/3 of the height of the eyes (Fig. 1D). In the description of *Neocalosoter* (by Girault and Dodd in Girault 1915), it is stated that the antennae in this genus are “inserted at mouth border”, i.e., below the lower margin of the eyes. However, the position of the toruli in *Pteropilosa lailarabanorum* is not unique within Cerocephalinae. *Sciatherellus*, *Dominocephala*, *Muesebeckisia*, and *Gnathophorisca* also have the antennae inserted above the lower margin of the eyes. *Muesebeckisia* and *Gnathophorisca* are easily distinguishable from *Pteropilosa* by their enlarged and conspicuously thickened mandibles. Furthermore, *Muesebeckisia* and *Gnathophorisca* share a fuscous band on the fore wing, which is absent in *Pteropilosa lailarabanorum*. The recently described fossil genus *Dominocephala* can be distinguished from *Pteropilosa lailarabanorum* by the elongate mandibles and the set of multiple setae on the lower facial process, both of which are not found in *Pteropilosa lailarabanorum*. *Dominocephala* also features a fuscous band on the fore wing not present in *Pteropilosa lailarabanorum*.

#### Etymology.

Named after Laila and Raban Ohlhoff, the grandchildren of the private donor.

### Tenuicornus gen. n.

Taxon classificationAnimaliaHymenopteraPteromalidae

http://zoobank.org/917FEC30-8E65-4848-982D-58982C4558C6

#### Type species.

*Tenuicornus dominicus* sp. n.

#### Material.

Female holotype, preserved in Lower Miocene Dominican amber (20-15 Ma). Holotype deposited in the amber collection of the Senckenberg Forschungsinstitut und Naturmuseum Frankfurt am Main, Germany, collection number SMF Be 2395.

#### Diagnosis.

Funicle of female 6-segmented and clavate with F1–F2 longer than wide and F3–F6 wider than long and distally broadening; scape elongate (1/3 of total antennal length) (Fig. 2D). Face deeply impressed with one distinct seta at each side of lateral margin of the impression anterior to mandibular articulation; seta long and thickened, slightly clavate in shape (Fig. 2C). Inner antennal process (iap) very thin and nail-like, located above level of toruli at upper margin of facial impression. Position of iap distinctly above ventral margin of eyes, on level with middle of eyes (Fig. 2C, D). Fore wing without a tuft of erect setae at parastigma. Wing disc bare, without setal bases. Marginal vein four times as long as stigmal vein (Fig. 2E). Prepectus large and triangular. Mesonotum almost completely polished (Fig. 2B). Exerted part of ovipositor long, approximately 1/4 of gaster length (Fig. 2A).

#### Etymology.

The first letters of the generic name *Tenui*- are derived from the Latin word “tenuis”, meaning “thin” or “sharp”. The last letters -*cornus* of the generic name are derived from the Latin word “cornus”, meaning “horn”. The generic name is male in gender and refers to the thin inner antennal process.

### Tenuicornus dominicus sp. n.

Taxon classificationAnimaliaHymenopteraPteromalidae

http://zoobank.org/8968FC81-D138-43FB-AA79-CD15E283D82C

#### Diagnosis.

See genus.

#### Description.

Female: total body length 2.07 mm; length of mesosoma 0.92 mm, of metasoma 1.02 mm. Body without metallic luster. *Head*: height 0.39 mm, width 0.38 mm. Face deeply impressed. Shape of head cuboid; mandibles not visible, hidden in facial depression. Margin of facial depression with rough surface and with a thickened seta on each side (length of setae 85 µm). Inner antennal process (iap) positioned distinctly above level of toruli; nail-like, 0.11 mm long, with basis wider than apex, orientated anteroventrally. Eyes large and egg-shaped, 0.25 mm high and 0.2 mm wide. *Antennae*: inserted at ventral margin of eyes; scape long and slightly curved, length 0.25 mm; pedicel length 75 µm, rather short and stout; funicle 6-segmented with first two funicular segments longer than wide (F1: length: 71 µm × width: 52 µm; F2: 68 µm × 60 µm) and F3-F6 wider than long, distinctly broadening distally, club-like (F3: 59 µm × 72 µm; F4: 60 µm × 74 µm; F5: 58 µm × 80 µm; F6: 52 µm × 86 µm). Clava egg-shaped: 97 µm long and 77 µm wide. *Wings*: fore wing long and slender (length 1.53 mm and width 0.55 mm); submarginal vein 0.57 mm long, marginal vein 0.38 mm long, postmarginal vein 0.11 mm long, stigmal vein 99 µm long; stigma slightly thickened and uncus visible. Wing disc bare, setal bases absent. Elongate admarginal setae present. Hind wing length 1.27 mm and width 0.27 mm, three hamuli present. *Mesosoma*: pronotum and anterior part of mesoscutum with slight traces of strigulate surface sculpture; mesonotum otherwise polished. Notauli foveolate. Length of pronotum 0.37 mm, length of mesoscutum 0.22 mm, length of mesoscutellum 0.22 mm; no frenal line. Axillae medially connected. Mesopleuron (in lateral view) height 0.29 mm, width 0.36 mm. Prepectus enlarged and triangular in shape (0.14 mm × 0.17 mm). Length of metanotum 39 µm; length of propodeum 72 µm. Propodeum without a median carina or plicae. *Legs*: coxae strong and stout. Femora and tibiae long and slender (Fe_1_: 0.45 mm; Fe_2_: 0.32 mm; Fe_3_: 0.41 mm; Ti_1_: 0.39 mm; Ti_2_: 0.49 mm; Ti_3_: 0.42 mm). *Metasoma*: petiole (Mt_1_) short and transverse, hardly visible. Metasomal tergites Mt_2_ to Mt_9_ smooth, Mt_2_: 0.28 mm, Mt_3_: 0.17 mm, Mt_4_: 0.15 mm, Mt_5_: 0.16 mm, Mt_6_: 0.11 mm, Mt_7_ 60 µm, Mt_8/9_: 71 µm. Ovipositor 0.23 mm (top view) exerted beyond end of gaster.

#### Taxonomic remarks.

The two genera that are most similar to *Tenuicornus* are *Neocalosoter* and *Cerocephala*. *Tenuicornus* runs to *Neocalosoter* in the key of Krogmann (2013) but differs from this genus in various features: *Tenuicornus* possesses a head that is deeply impressed (convex in *Neocalosoter*), the dark transverse bands on its fore wing are absent, and the propodeum has no traces of a median carina or plicae (Fig. 2B). The tentative similarity between *Tenuicornus* and *Cerocephala* is based on the deeply impressed face. However, *Tenuicornus* differs from *Cerocephala* by the absence of a tuft of setae on the parastigma on the fore wings (Fig. 2E), one of the most distinct characters for the generic classification of Cerocephalinae. *Tenuicornus* further differs from *Cerocephala* by the shape and position of the inner antennal process. In *Tenuicornus*, the process is shaped like a nail and positioned above the level of the toruli at the upper margin of the facial depression (Fig. 2C). In *Cerocephala*, the process is broader and stouter and positioned at or slightly below the level of the toruli. Furthermore, *Cerocephala* is described to have all funicular segments longer than wide (Westwood 1832), while in *Tenuicornus* only the first two funicular segments are longer than wide. The funicular segments three to six are wider than long (Fig. 2D).

#### Etymology.

The name *dominicus* is derived from the amber deposit in which the fossil was found.

## Discussion

*Pteropilosa lailarabanorum* features a variety of characters that are unique within Cerocephalinae. The most striking of these characters is the wing pilosity (Fig. 1B). In some genera of Cerocephalinae, such as *Cerocephala* (in part), *Neosciatheras*, and *Gnathophorisca*, there are still setal bases present, but no distinct pilosity. It is plausible to assume that pilose wings represent the ground plan character state for Cerocephalinae and that the pilosity has been reduced in crown-group lineages. This loss might already have happened before the Miocene. While *Dominocephala* still possesses setal bases, they are completely lost in *Tenuicornus* (Fig. 2E). Also, the presence of a distinct frenal line in *Pteropilosa* (Fig. 1C) most likely represents a plesiomorphic character state (see Krogmann and Vilhelmsen 2006) and has consequently been reduced in Miocene and extant Cerocephalinae. The same seems to apply to the strong surface sculpture of the mesonotum of *Pteropilosa* (Fig. 1C). In all other cerocephaline taxa, the mesonotum is polished or only partly sculptured.

Since both fossils were found without syninclusions (in contrast to *Dominocephala*), it is impossible to speculate about the host species of the newly described genera. However, the short and stout ovipositors and the overall similarity of body size and appearance with extant taxa of Cerocephalinae suggest that both genera are, like all known extant representatives, parasitoids of endophytic beetles.

Although all three known fossils are expected to be parasitoids of endophytic beetles, there are already hints of differential host specialization. The ovipositor of *Tenuicornus* is further exserted beyond the end of the gaster than in the other two fossils, which might serve as an indicator that this genus has a different host biology than *Dominocephala vibrissae* and *Pteropilosa lailarabanorum*.

All three known cerocephaline fossils are females. This is most likely connected to the association with wood-boring beetles, which represent the vast majority of known cerocephaline hosts. Females visit trees for oviposition and have a much higher chance of being trapped in resin than males. Members of the family Ptinidae, a common host group of Cerocephalinae (Adarkwah et al. 2014), are closely associated with resin-producing trees and rank among the four most abundant beetle families in Baltic amber (Bukejs and Alekseev 2015). Other known hosts for Cerocephalinae are ambrosia beetles (Curculionidae: Platypodinae), which are diverse and common inclusions in Dominican amber (Peris et al. 2015) and were found as syninclusions of *Dominocephala vibrissae* (Krogmann 2013).

*Tenuicornus* has a very conspicuous single thickened and enlarged seta on each side of the facial impression (Fig. 2C). Similar structures were found on the face of *Dominocephala vibrissae* and the extant *Choetospilisca tabidoides* Hedquist, 1969. In *Dominocephala vibrissae*, a row of elongate setae extends dorsally from the lower facial process. *Choetospilisca tabidoides* features a set of long setae that cover the area between the toruli and the mandibles. Although the amount of setae varies, the position is similar, i.e., all setae are positioned at the most frontal position of the face. In this prominent position, they might serve as a sensory organ for the location of hosts in wood or other plant parts.

The description of *Pteropilosa* pushes the minimum age of Cerocephalinae from the Miocene to the Eocene and hints to an earlier host shift to endophytic beetle parasitoids than previously thought (Krogmann 2013). The occurrence of two morphologically distinct genera in Dominican amber shows that Cerocephalinae had already significantly diversified by the Miocene. Future examination of unstudied material and understudied amber Lagerstätten might further change or complement our view on the evolution of Cerocephalinae as well as of other Chalcidoidea groups.

## Supplementary Material

XML Treatment for Cerocephalinae

XML Treatment for Pteropilosa

XML Treatment for Pteropilosa lailarabanorum

XML Treatment for Tenuicornus

XML Treatment for Tenuicornus dominicus
